# A feasibility randomized trial of an identity-based physical activity intervention among university students

**DOI:** 10.1080/21642850.2019.1600407

**Published:** 2019-04-10

**Authors:** Cassandra J. Husband, Joan Wharf-Higgins, Ryan E. Rhodes

**Affiliations:** Behavioural Medicine Lab, School of Exercise, Physical, and Health Education, Faculty of Education, University of Victoria, Victoria, Canada

**Keywords:** Physical activity, Identity, Feasibility, Controlled trial, University students

## Abstract

INTRODUCTION: Exercise identity has considerable evidence as a correlate of physical activity (PA), but almost no research has focused on intervention. Theory suggests identity may be formed through indirect means of motivated behaviour change over time or through direct targeting of identity related antecedents. Using a parallel, single blind design, the purpose of this study was to explore the feasibility (recruitment, retention, and satisfaction) of these two types of interventions (indirect, direct) to increase exercise identity and subsequent PA. METHODS: Participants between the ages of 18–25 who were not meeting PA guidelines were recruited from the University of Victoria, and randomized at a 1:1 ratio to an indirect or direct intervention group. The indirect intervention group received information on the benefits of PA and behaviour change techniques such as planning. The direct intervention group received the same information, with the addition of identity-specific information. Intervention materials were delivered bi-weekly for 6 weeks. Feasibility and participant satisfaction at the study end-point were assessed using mixed methods, and both PA change and exercise identity change were assessed via self-report. RESULTS: Twenty participants were randomized to the direct or indirect intervention group (10 each), with 18 participants completing full study protocol. The recruitment rate was 26% and retention was 90%. Mean scores from the satisfaction survey (five-point scale) were high for both groups (indirect *M* = 2.69, SD = 0.62; direct *M* = 2.83, SD = 0.40). Both intervention groups increased their PA (*η^2^* = 0.25), and exercise identity levels (*η^2^* = 0.43) across six weeks. DISCUSSION: High feasibility ratings, both through retention, and survey and interview data show that the study could be extended to a full-scale RCT. Modifications to recruitment including oversampling to account for low recruitment rates may be useful. No adverse events were reported.

Regular physical activity (PA) has been reliably linked to both physical and mental health benefits (Lee et al., 2012; Rebar, Stanton, Geard, Short, & Duncan, 2015; Rhodes, Janssen, Bredin, Warburton, & Bauman, 2017; Warburton, Nicol, & Bredin, 2006). Unfortunately, worldwide estimates suggest that up to 43% of adults are not meeting PA guidelines (Hallal et al., 2012). One particularly at risk group are university students, who show a marked drop in physical activity from high school (Bray & Born, 2004). Thus, university students are a key target for PA interventions.

Currently, the majority of interventions follow a social cognitive framework, following recommendations from theories such as the Social Cognitive Theory (Bandura, 1998), the Theory of Planned Behaviour (Ajzen, 1991), or the Transtheoretical model (Prochaska and DiClemente, 1982). Although these models/theories differ in their naming of key constructs, they all focus on intervening upon expectations of utility, social norms, and perceptions of capability (Fishbein, Triandis, Kanfer, Becker, Middlestadt, & Eichler, 2001). As an extension of these models, self-regulation techniques such as goal setting or self-monitoring are often employed within PA interventions (Michie, Abraham, Whittington, McAteer, & Gupta, 2009). Recent meta-analytic work indicates PA interventions employing these models yield a small effect size (*d = *0.27) (Rhodes, Janssen, Bredin, Warburton, & Bauman, 2017).

Given the modest impact of PA interventions in the social cognitive tradition, there have been calls to extend intervention approaches to other types of frameworks (Hagger & Chatzisarantis, 2014; Rebar et al., 2015; Rhodes, 2017; Williams & Evans, 2014). Some theorists have argued that the social cognitive tradition focuses almost exclusively on reflective processes, which generate behavioural decisions that are based on knowledge about facts and values (Strack & Deutsch, 2004). These authors and others (Gardner, De Bruijn, & Lally, 2011; Rebar et al., 2016; Rhodes, 2017; Rhodes, Kaushal, & Quinlan, 2016; Stryker & Burke, 2000; Williams & Evans, 2014) suggest that reflexive processes, those that occur quickly or on impulse, may also impact PA. The inclusion of reflexive processes such as implicit attitudes, automatic affective associations, habit, or identity may provide a more complete picture of PA performance and increase the effectiveness of interventions (Rebar et al., 2016; Ries, Hein, Pihu, & Armenta, 2012; Sheeran, Gollwitzer, Bargh, Gollwitzer, & Bargh, 2013). The focus of the current study is on exercise identity within this context.

Exercise identity is the self-categorization of oneself into a profile of a regular exerciser. This self-categorization is thought to act as its own reflexive, self-regulating mechanism of motivation, independent of other reasons for performing the behaviour such as outcome expectations (Berry, Strachan, & Verkooijen, 2013; Rhodes et al., 2016; Stets & Burke, 2000; Stryker & Burke, 2000). The reflexive aspect of identity refers to the premise that identity motivates behaviour mainly to reduce negative affect when there is misalignment between one’s self-categorization and observed behaviour (Burke, 2016; Stryker & Burke, 2000). Thus, as one grows less concordant between identity and behaviour, the dissonance prompts motivation to reduce the discrepancy (Festinger, 2016; Stets & Burke, 2000). Identity is a promising construct due to its reflexive composition, its maintenance level capacity [maintenance mechanisms are thought to aid in the long term sustainability of a behaviour (Kwasnicka, Dombrowski, White, & Sniehotta, 2016)], and its capacity to moderate intentions into behaviour by acting on self-regulatory processes (Rhodes, Gray, & Husband, 2018). Thus, exercise identity is considered both an outcome of sustained behaviour as well as a determinant of behaviour.

The relationship between exercise identity and PA participation has a medium effect size (*r = *0.44) according to a recent meta-analysis (Rhodes et al., 2016), suggesting a potentially critical construct in the understanding PA. Still, this recent review found that almost all evidence for identity in the PA domain was from observational studies. Intervention research is needed to explore the techniques that modify identity and its effect on PA change.

According to several theories, there are two conceivable routes through which identity change may occur (Figure 1). First, exercise identity may develop as a natural consequence of changed motivational characteristics (Kendzierski, Furr, & Schiavoni, 1998), self-regulation abilities (Stadler, Oettingen, & Gollwitzer, 2009), and reflections upon changed PA behaviour (Bem, 1972; Burke, 2006; Kendzierski, Furr, & Schiavoni, 1998; Rhodes, 2017). This ‘indirect’ route generally suggests that identity may develop over time as a function of standard interventions such as those employed in the social cognitive tradition.

**Figure 1.**
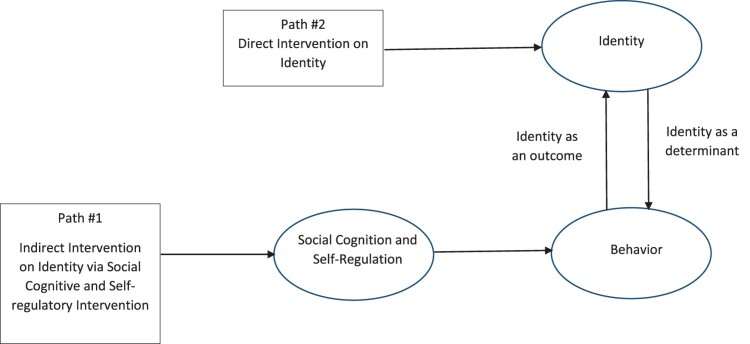
Logic model of relationship between exercise identity and exercise behaviour.

The second route through which identity change may occur is through direct targeting of identity-related concepts (Michie et al., 2013). In this route, the interventionist may accelerate the lengthy process of sustained motivational and behavioural change that lead to exercise identity development via behaviour, and instead expedite the identity formation process by also targeting identity directly. The Self-Definition Model (SDM) (Kendzierski, Furr, & Schiavoni, 1998), PRIME Theory (West, 2009), and the Multi-Process Action Control Framework (M-PAC) (Rhodes, 2017) all highlight this route of intervention. Specifically, the SDM (Kendzierski, Furr, & Schiavoni, 1998) postulates that the extent to which others in someone’s social world acknowledge the self-definition, and mention their engagement in the activity, will impact the development of an identity. PRIME Theory (West, 2009) indicates that identity is strengthened by clear rules of enactment, while the M-PAC highlights the importance of prioritizing and expressing one’s identity to others (Rhodes, 2017).

The purpose of this study was to examine the feasibility of an exercise identity intervention across six weeks using these two potential routes to identity formation. A feasibility study is performed to establish whether an intervention is appropriate for further testing on a grander scale (Bowen et al., 2009). It was hypothesized that feasibility (recruitment, retention, and satisfaction) would be satisfactory; meaning recruitment rates of 35–40% (based on similar designs and samples from Arigo, 2015; Bray et al., 2011; Canning, Allen, Dean, Goh, & Fung, 2012; Kwan, Faulkner, & Bray, 2013; Skår, Sniehotta, Molloy, Prestwich, & Araújo-Soares, 2011; Van Oort, Tupper, Rosenberg, Farthing, & Baxter-Jones, 2013), retention of 80–100% (Jackson & Waters, 2005), and a score of ≥ 2.5 on the satisfaction and evaluation questionnaire. The secondary purpose of this research was to examine preliminary trends in PA and exercise identity change. It was hypothesized that both intervention groups will show trends of increased PA but the direct intervention may show larger trends in identity than the indirect route due to direct targeting and discussion of exercise identity (Rhodes, 2017).

## Materials and methods

The study featured a mixed method, single blind, parallel design, randomized feasibility trial, in which the study coordinator was aware of group assignment but the participants were not. The study followed Consort guidelines for pilot and feasibility trials (Eldridge, Chan, Campbell, Bond, & Hopewell, 2016).

### Eligibility criteria

Eligible participants were undergraduate students at a University in Western Canada, ages 18–25. Participants were excluded if they were currently meeting PA guidelines (150 min of MVPA per week), or were not seeking to increase their PA participation. Participants self-screened for this study using the above inclusion criteria.

### Procedures

Recruitment was achieved using posters and telecaster advertisements across the University campus, as well as personal recruitment through classroom visits. The first author recruited participants in September and October 2017, as well as January and February 2018, and stopped recruiting when 20 participants had been randomized into the trial. All participants were entered into a draw for a $50 grocery gift card. Once a person expressed interest in the study, a baseline appointment was booked at the University, in which the participant gave consent, completed baseline measures, and was subsequently randomized by the first author at a 1:1 ratio to either the indirect intervention or direct identity intervention group. Randomization used a random number sequence generator, and participants were identified thereafter using participant numbers. After random assignment, the participant was presented with the appropriate intervention materials one-on-one, and scheduled the next session. This proceeded for four sessions (occurring bi-weekly). The last session included a follow-up questionnaire with satisfaction and evaluation measures, and an invitation for an exit interview which was scheduled during the coming 7 days. All participants were invited to participate in the exit interview. There were no changes to methods after the commencement of the study.

### Feasibility measures

Recruitment rate was calculated by dividing the participants scheduled for a baseline by the number of participants who signed up as ‘interested’ in the study. Retention was calculated at the six-week study endpoint by dividing a number of completed participants by the number of baseline participants. Satisfaction was evaluated via a 9-item satisfaction and evaluation questionnaire, adapted from the satisfaction questionnaire from Forbes, Blanchard, Mummery, and Courneya (2015) and Rhodes et al. (Quinlan, Rhodes, Blanchard, Naylor, & Warburton, 2015; Rhodes, Naylor, & McKay, 2010). Sample questions included ‘how interesting was the information provided in the workbooks?’ and ‘how effective was the material delivered by the researcher?’ The satisfaction and evaluation questionnaire was on a 4-point scale in which 1 was *least satisfied* and 4 was *most satisfied*. The exact wording of options 1 through 4 changed depending on the question. Similar to Forbes et al. (2015), participant satisfaction was additionally assessed through optional exit interviews at the end of the intervention period (six weeks). The questionnaire included open ended questions designed to understand satisfaction with the overall content and delivery of the intervention. Sample questions included ‘how did you feel about the study?’ and ‘what would you change about the study?’. Exit interview questions can be found in Appendix 1.

### Secondary outcomes measures

PA participation was assessed using the Godin Leisure-Time Exercise Questionnaire (GLTEQ) (Godin & Shephard, 1997). It has been shown to be valid and reliable in different settings, populations and countries (Gionet & Godin, 1989; Godin & Shephard, 1997), with test-retest reliability among healthy adults recently demonstrated at *k* = 0.65, which the authors deemed satisfactory (Amireault & Godin, 2015). To create a leisure score index (LSI), bouts of vigorous PA were multiplied by nine, and added to the number of bouts of moderate PA multiplied by five (Godin & Shephard, 1997), which is in accordance with Canadian PA guidelines (Canadian Society for Exercise Physiologists, 2018). According to Amireault and Godin (2015), a ‘sufficiently active’ person has an LSI score greater than or equal to 24. Exercise identity was assessed using the exercise identity scale (Anderson & Cychosz, 1994), which is a nine-item questionnaire on a five-point likert scale from 1 *(strongly disagree)* to 5 *(strongly agree)*. Higher scores on the exercise identity scale indicated a stronger exercise identity. Anderson and Cychosz (1994) demonstrated test-retest reliability for this scale of 0.93 among healthy university students which are commensurate with the reliability of this study at baseline (*α* = 0.92) and six weeks (*α* = 0.90).

### Intervention content

The details of the intervention materials were derived from prior intervention literature (see Table 1 for details). The indirect group materials included information on the benefits of PA, goal setting, planning, and self-monitoring. The direct intervention group materials contained the same information and techniques as the indirect group, as well as additional identity-specific content. For the direct intervention group, session 1 brought awareness to the concept of identity, and emphasized the importance of skill, enjoyment, and variety of PA participation. Session 2 included an activity on prioritizing PA (including what the participant spends their money, time, and effort on), setting rules around PA participation, and developing positive self-talk related to exercise identity (i.e. ‘I am an exerciser, therefore I will … ’). Session 3 discussed the environment, and asked the participants to use symbolic representation to ‘up’ their feelings of being an exerciser. The verbal dialogue included discussion around sport participation (and by extension, trying a variety of activity types), and sport ownership throughout the six weeks. Full intervention materials for both groups can be found in Appendix 2.

**Table 1.** Behaviour change techniques (BCT) utilized in intervention content.

Group	BCT	Session component
*Indirect Intervention*		
** **	1.1 Goal setting (behaviour)	Setting SMART goals
** **	1.2 Problem solving	Barrier identification
** **	1.4 Action planning	Creating a detailed PA plan
** **	1.5 Review behavioural goal(s)	Each week, goals were reviewed and revised
** **	2.3 Self-monitoring of behaviour	Self-monitoring explicitly discussed
** **	5.1 Information about health consequences	Physical health consequences explicitly discussed
** **	5.6 Information about emotional consequences	Emotional consequences explicitly discussed
** **	8.7 Graded tasks	SMART goals including reasonable goals for individual’s current fitness level
*Direct Intervention*		
** **	1.6 Discrepancy between current behaviour and goal	Activity on ideal self vs actual self
** **	7.1 Prompts/cues	Environmental cuing explicitly discussed
** **	8.3 Habit formation	Habit formation explicitly discussed
** **	12.1 Restructuring the physical environment	Changing one’s environment explicitly discussed
** **	12.4 Distraction	Discussed when brainstorming ways to make physical activity fun
** **	12.5 Adding objects to the environment	Discussed during section on changing the environment
** **	13.2 Framing/reframing	Cognitive restructuring of physical activity beliefs
** **	13.3 Incompatible beliefs	Drawing attention to the fact that a participant exercises but does not consider themselves ‘an exerciser’
** **	13.5 Identity associated with changed behaviour	Discussion around creating a new identity surrounding exercise, including changing dress, social media presence, etc
** **	15.4 Self-talk	Positive self-talk regarding being an exerciser (ex ‘I am an exerciser therefore I will … ’)

Note: Direct intervention group received the indirect intervention BCTs as well as the direct intervention group BCTs; see Appendix 2 for full intervention materials

### Analysis plan

Participant recruitment rate was compared to studies of similar design to assess feasibility including other six-week feasibility studies and feasibility studies with a university population (Arigo, 2015; Bray et al., 2011; Canning et al., 2012; Kwan et al., 2013; Skår et al., 2011; Van Oort et al., 2013). The average recruitment rate of these studies was 37%, thus we deemed a recruitment rate of 35–40% to be successful. A 80–100% retention rate is indicative of a strong trial (Jackson & Waters, 2005). For satisfaction ratings, mean and standard deviations were calculated for the quantitative satisfaction and evaluation questionnaire. Given that scores of 1 (ex. not helpful, did not use the tools) and 2 (ex. Somewhat helpful, used a little bit of the tools) indicated levels of dissatisfaction, and 3 (ex. Quite helpful, used a bit of the tools) and 4 (ex. Extremely helpful, used a lot of the tools) indicated some sort of satisfaction, a mean score of ≥ 2.5 was deemed acceptable for recommendation for a full RCT. The interviews were transcribed verbatim and analysed using NVivo 11 (QSR International) software to identify common themes or valences (i.e. similar recommendations for change, overall positive/negative comments) within and between participant groups. Both quantitative and qualitative satisfaction evaluations followed recommendations from previous literature (Linnan & Steckler, 2002; Moore et al., 2015), such as ensuring that quantitative and qualitative analyses build on each other, as well as analysing process data before trial outcomes are known. Collectively, if the direct intervention has acceptable feasibility ratings including recruitment (comparable participation rates), retention (80–100% retention), and satisfaction (average of ≥ 2.5 on the satisfaction and evaluation questionnaire, and generally positive qualitative feedback), a full-scale trial would be recommended.

Secondary outcome measures were analysed using SPSS version 23.0 for Windows. Effect size conventions followed Cohen’s (1992) recommendations: small (*d* = .20), moderate (*d* = .50), and strong (*d* = .80). Mixed-design analysis of variance (ANOVA) was used to determine both the main effect and interaction effect of the direct intervention compared to the indirect identity intervention on both PA and exercise identity scores. Confidence intervals for mean difference scores for both PA and exercise identity data were also calculated. Confidence intervals that cross zero are considered not convincing of a meaningful difference in the data (Field, Field, & Miles, 2013).

## Ethics Statement

This study was approved by the Human Research Ethics Board at the University of Victoria (protocol number 17-309).

## Results

### Participants

**Figure 2.**
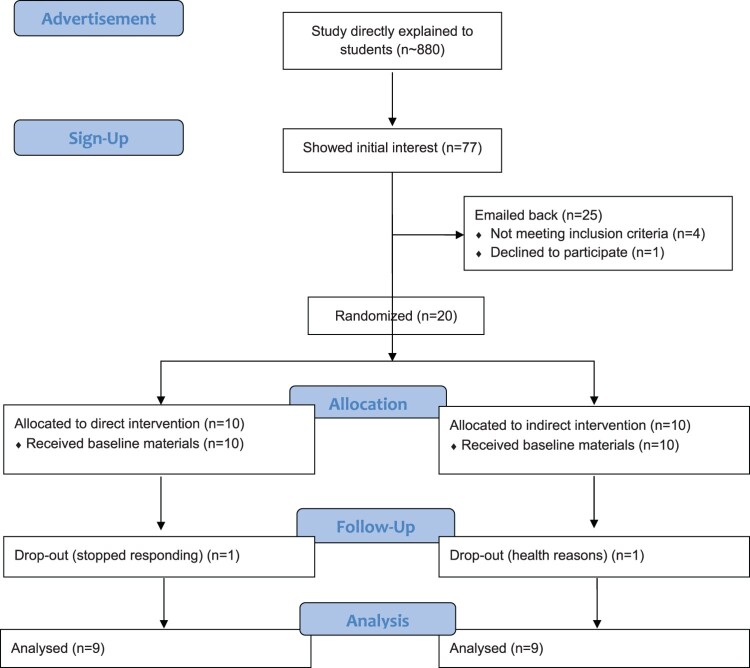
CONSORT flow diagram of sign-ups, allocation, participant progress, and analysis.

A Consort flow diagram (Figure 2) is presented below with full details of participant trajectory through the study (Schulz, Altman, Moher, & Group, 2010). Twenty participants (10 in each group) were recruited and randomized. This number is aligned with previous feasibility trials conducted over a six week period (Arigo, 2015; Canning et al., 2012; Van Oort et al., 2013). Baseline participant characteristics are presented in Table 2. The mean age of participants was 21.33 (*SD* = 2.30) and they were predominantly female (13/18, 72.2%). Participants were well distributed across the year in university (first to the sixth year) as well as faculty membership. At baseline, participants had a mean LSI of 26.14 (*SD* = 19.33), indicating a ‘sufficiently active’ sample (LSI score ≥ 24) (Amireault & Godin, 2015). As well, baseline exercise identity scores (*M* = 3.12, *SD* = 0.92) indicate a sample that is in the ‘medium’ exercise identity range (*M* = 2.40–3.60). Similar to work by Strachan and Brawley (2008) the researchers used a tertile split to group potential participants into the low, medium, and high identities based on their exercise identity questionnaire scores obtained from previous research. However, because there were not enough participants to create statistically significantly different groups, the researchers instead split the range of possible mean scores on the exercise identity questionnaire into three equal portions. Low identity corresponded to a mean score of 1.0–2.3, medium identity corresponded to a mean score of 2.4–3.6, and high identity corresponded to a mean score of 3.7–5. The exercise identity scale has 9 items measured on a five point scale of 1*(strongly disagree)* to 5*(strongly agree)* with 3 being *(neutral)*.

**Table 2.** Baseline characteristics of participants: mean (SD).

Characteristic	Overall (*n* = 18)	Indirect (*n* = 9)	Direct (*n* = 9)
Age	21.33 (2.30)	22.11 (2.42)	20.56 (2.01)
Leisure Score Index	26.14 (19.33)	27.00 (18.21)	25.28 (21.47)
Exercise Identity Score	3.12 (0.92)	2.86 (0.59)	3.38 (1.14)
Year in University	3.22 (1.73)	3.33 (1.94)	3.11 (1.62)

### Feasibility

Of the seventy-seven people who expressed interest in the study, twenty-five replied to the email for the baseline assessment request. Four participants were excluded due to not meeting inclusion criteria, and one participant declined to participate upon receiving more information about the study, yielding a recruitment rate of 26% (20/77).

20 participants were randomized to indirect or direct intervention groups, of which 18 completed the study protocols – a 90% retention rate. Two participants dropped out of the study after it had begun; one between 2 and 4 weeks, and one at 6 weeks.

For the indirect identity intervention group, mean satisfaction was 2.69 (SD = 0.62), with workbook satisfaction (*M* = 2.69, SD = 0.87) ranking the same as counselling session satisfaction (*M* = 2.69, SD = 0.43). For the direct identity intervention participants, mean satisfaction was 2.83 (SD = 0.40), with intervention materials satisfaction (*M* = 2.81, SD = 0.50) ranking similar to counselling session satisfaction (*M* = 2.86, SD = 0.44).

The direct intervention group participants rated six of the nine questions in the satisfaction and evaluation questionnaire more positively than did indirect intervention group participants, with effect size differences ranging from *d* = 0.15–0.48. Two of the questions yielded the same mean score. Two questions were rated higher by the indirect intervention group participants than the direct intervention group participants, with effect size *d* ranging from −0.42 to −0.67 (Table 3).

**Table 3.** Satisfaction and evaluation questionnaire results.

Satisfaction and Evaluation Questions	Overall (*n* = 18)	Indirect Intervention (*n* = 9)	Mean (SD)	Direct Intervention (*n* = 9)	Mean (SD)	Effect size d
Information provided in workbooks: *Quite or extremely interesting*	14/18 (77.8%)	6/9 (66.7%)	2.67 (0.87)	8/9 (88.9%)	3.00 (0.50)	0.48
Learned anything new from the workbooks: *Learned quite a bit or a lot*	8/18 (44.4%)	3/9 (33.3%)	2.22 (0.97)	5/9 (55.65)	2.67 (0.71)	0.54
Used the tools and strategies provided in the workbooks: *Used quite a bit or a lot*	11/18 (61.1%)	6/9 (66.7%)	2.89 (1.05)	5/9 (55.6%)	2.56 (0.53)	−0.42
Tools provided in workbooks helped increase physical activity: *Helped quite a bit or a lot*	11/18 (61.1%)	6/9 (66.7%)	3.00 (1.12)	5/9 (55.6%)	3.00 (0.87)	0.00
Learn any new information working with the researcher: *Learned quite a bit or a lot*	10/18 (55.6%)	4/9 (44.4%)	2.44 (0.53)	6/9 (66.7%)	2.89 (0.78)	0.68
Used the tools discussed during counselling sessions: *Used quite a bit or a lot*	13/18 (72%)	5/9 (55.6%)	3.00 (1.00)	8/9 (88.9%)	3.22 (0.67)	0.26
Counselling sessions helped to increase PA: *Quite helpful or extremely helpful*	16/18 (88.9%)	7/9 (77.8%)	3.22 (0.83)	9/9 (100.0%)	3.22 (0.44)	0.00
Amount of exposure to counselling sessions: *Good amount*	17/18 (94.4%)	9/9 (100%)	2.00 (0.00)	8/9 (88.9%)	1.89 (0.33)	−0.67
Effectiveness of material delivery: *Quite effectively or extremely well delivered*	16/18 (88.9%)	8/9 (88.9%)	3.33 (0.71)	8/9 (88.9%)	3.44 (0.73)	0.15

Note: ‘Amount of exposure to counselling sessions’ ranked from 1 *(not enough)* to 3 *(too much),* with 2 being *good amount*

Eleven participants opted in for the exit interview: five from the indirect intervention group and six from the direct intervention group (Table 4). For the indirect intervention group participants, the intervention was described using positive words such as ‘good’, ‘loved’, or ‘liked’ for 4/5 participants. The majority of participants (3/5 participants) cited study features such as the interactivity, or the focus on PA, as their favourite part of being in the study. Interestingly, 3/5 participants in the indirect intervention group did not have a response when asked about their least favourite part of the study, although those who did respond (2/5 participants) agreed that their least favourite aspect was the guilt they felt when they failed to meet their goals. Two of the five participants did not have an answer for ‘what would you change about the study?’ however, all three who answered gave constructive criticism, saying that the material was delivered well, but suggested changing study logistics such as increased use of technology including online check-ins or digital access to materials. One participant was not satisfied with the study overall, saying in his interview

So, what I was expecting was like “you should be doing this, this is like, let’s do exactly like step 1 step 2 step 3 if you want to up your activity level this much, these are the things you should be doing” you know, a lot more structured rather than set your own goals and see if you can follow through on those.

This participant recommended adding more structure to the intervention for people who are ‘not super determined to really get all the physical activity they can’.

**Table 4.** Results from satisfaction and evaluation exit interviews.

Satisfaction and evaluation questions	Indirect intervention	Sample quote	Direct intervention	Sample quote
How did you feel about the study?	1/5 neutral 4/5 positive	Oh my gosh I actually loved it. Like I, cause I actually like had an increase (in physical activity). And I liked that it wasn’t focused around like performance	6/6 positive	I really enjoyed like the identity – exercise identity piece of it, like that was kind of what hit home the most
What was your favourite part?	1/5 N/A 1/5 liked increasing PA 3/5 liked study features	Just getting back into it and knowing that I could. Cause I think it can be a very mental thing sometimes where you sort of psych yourself out before you’ve even tried it, so yeah I think that I came into this feeling very, like it was going to be a huge challenge, and obviously it was in some aspects but I think that I made it to be more of a challenge in my head than it was in reality.	1/6 liked study features 1/6 liked increasing PA 4/6 liked interaction with researcher and counseling sessions	Definitely just like the personal interaction and talking about, just having somebody to talk to about all the things that are going on. I don’t really get that anywhere else, so it’s good to take off a little bit of weight there … emotional weight
What was your least favourite part?	3/5 N/A 2/5 goal related guilt	During the weeks when I didn’t exercise I felt kind of like guilty or embarrassed to talk about it. Because this is an exercise study and I felt really lazy and like couch potato, not because of you but just because, it was embarrassing to report on it that I hadn’t been to the gym.	1/6 N/A 5/6 goal related guilt	Probably just in the beginning when things weren’t working before I had developed some strategies I guess? Cause like it’s nice to be able to check in with you and actually say ‘yes I did meet my goals’ it’s kind of disappointing to have to say no, it sucks, but it’s part of the learning curve I guess.
What would you change?	2/5 N/A 3/5 intervention logistics, intervention structure, materials	Something more structured time wise would probably be more wide reaching in its effects, rather than … and probably apply to more people rather than letting people just sort of forget about it.	1/6 N/A 5/6 intervention logistics, intervention structure, materials	Maybe the timing, like when it was. It would have been easier at the beginning of the semester, it would have been good to get into the rhythm when the semester started instead of doing it at the end when all hell was breaking loose.

For the direct intervention group, the intervention was described using positive words such as ‘enjoyed’, ‘really liked’ or ‘good’ for 6/6 participants interviewed. The group differed from the indirect intervention group in that overall (4/6 participants), their favourite parts were interactions and counselling sessions with the instructor. However, similarly to the indirect intervention group, 5/6 participants reported that their least favourite part of the study was goal related guilt or disappointment. Additionally, the dialogue around what the participants would change about the study was largely the same as the indirect intervention group. One of six participants did not have recommendations for change, while 5/6 participants referenced things like changing the take-home-materials to make them feel less ‘disconnected’ as well as more ‘in your face’. Additionally, one participant who joined late in the school term recommended changing the timing of the study, stating she would rather have started earlier to have more time to get into a routine before the semester ended.

### Adverse events

There were no adverse events reported due to participation in this study, however, one of the participant drop-outs was due to personal health reasons.

### Secondary outcome measures

There was a large main effect (*η^2^*^ ^= 0.25) of intervention on LSI scores from baseline to 6 weeks, however, the interaction effect size was small (*η^2^* = 0.02). The change score for the indirect intervention group was 8.11 [95% CI −7.39–23.61], and the direct intervention group was 12.61 [95% CI −1.39–26.61] (Table 5).

**Table 5.** Secondary outcome measures at baseline and 6 week.

Characteristic	Indirect (*n* = 9)			Direct (*n* = 9)			Main effect		Interaction effect	
	Baseline	6 weeks	Change score [95% CI]	Baseline	6 weeks	Change score [95% CI]	F-value	Partial Eta Squared	F-value	Partial Eta Squared
Mean Leisure Score Index (SD)	27.00 (18.21)	35.11 (14.76)	8.11 [−7.39–23.61]	25.28 (21.47)	37.89 (26.88)	12.61 [−1.39–26.61]	5.23	0.25	0.63	0.02
Mean Exercise Identity score (SD)	2.86 (0.59)	3.54 (0.69)	0.68 [0.23–1.13]	3.38 (1.14)	3.83 (0.80)	0.44 [−0.16–1.05]	11.90	0.43	0.52	0.03

At baseline, the exercise identity scores between the indirect and direct identity intervention groups were different (*d = *0.60), with the direct intervention group participants scoring higher. There was a large main effect (*η^2^*^ ^= 0.43) on exercise identity scores from baseline to 6 weeks, however, the interaction effect size was small (*η^2^*^ ^= 0.03). The change score for the indirect intervention group was 0.68 [95% CI 0.23–1.13], and the direct intervention group was 0.44 [95% CI −0.16–1.05].

## Discussion

The purpose of this research was to test the feasibility of two potential identity interventions across six weeks among a university undergraduate sample. Recruitment rates were 26%, which was lower than the predetermined recruitment cut-off of 35% to 40%. Still, this was within a reasonable range when comparing to other studies, where recruitment rates ranged from 23% to 48% (Arigo, 2015; Canning et al., 2012; Van Oort et al., 2013). While these comparator studies did not target healthy university students, they did employ a similar six week design. To compare recruitment to other university samples, Bray et al. (2011) had a 61% recruitment rate from five Canadian universities for their print-mediated PA intervention, although they did not include eligibility criteria of not meeting PA guidelines (<150 min of moderate to vigorous PA per week). Another university-based study (Kwan et al., 2013) evaluating a website-delivered PA intervention recruited 91 of 198 students living in an on-campus residence (46% recruitment rate). Finally, using mass email recruitment for their study examining an online planning intervention designed to increase PA levels, Skår et al. (2011) yielded a 10% recruitment rate (1273/13000 students). The above comparisons indicate that recruitment was acceptable in comparison to other six-week designs, but low in comparison to other studies targeting university samples, and low compared to the predetermined cut-off for this feasibility study.

One possible explanation for this recruitment rate is that people were not interested in adding to their already busy schedules due to high scholastic demands (Gyurcsik, Bray, & Brittain, 2004). Additionally, given that university students make up a large portion of research participants (Peterson & Merunka, 2014), students in general may be fatigued from previous research advertising and participation. In the future, we recommend over-sampling in order to obtain the desired number of participants. Mass email recruitment is one viable option. One-on-one referrals such as from campus health services may also serve to bolster recruitment rates. Media blasts from graduate secretaries, as well as though social media outlets may be helpful, especially at the beginning of university semesters (September and January). With a larger budget, monetary compensation more commensurate with expended time and effort could be offered in exchange for participation (over and above the $50 grocery gift card draw used in this research).

It was hypothesized that retention rates would be high for this study. Retention rates of 80–100% are indicative of a strong trial (Jackson & Waters, 2005). This study yielded a 90% retention rate, which is commensurate with feasibility studies with 6-week designs, including 100% (Van Oort et al., 2013), 90% (Canning et al., 2012), and 100% (Arigo, 2015). In comparison to other studies with university samples, a 90% retention rate is high. Bray et al. (2011) reported a 27% retention rate in a six week study, Kwan et al. (2013) reported a 33% retention rate in an eight week study, and Skår et al. (2011) reported a 53% retention rate in a two month study The lower retention rates seen for larger scale trials with university students may indicate a need to over-sample in order to mitigate potential drop-outs.

It was hypothesized that satisfaction scores would be high overall and our results supported this assertion. Scores from the satisfaction and evaluation questionnaire were within the pre-determined acceptability range of ≥ 2.5. The indirect identity intervention group satisfaction appeared to be lower than direct intervention group satisfaction, with a small effect size difference (*d = *0.27). This may support the notion that novel identity material was more satisfactory for participants than more standard social cognitive information. Furthermore, in the satisfaction and evaluation questionnaire, six of nine questions yielded higher responses for the direct group, either in the mean score or percentage of participants rating it highly. This suggests that the direct identity intervention group participants were more satisfied with the intervention than were the indirect group participants.

The exit interview results were largely the same between groups, with the exception of ‘favorite aspects’ of the study. Indirect intervention group participants cited specific study features, or their increased PA participation as favourite parts, while direct identity intervention group participants spoke instead about aspects involving meeting and spending time with the instructor. This speaks to the potentially more engaging material with the identity intervention, given that both groups received equal face-to-face time.

Overall, participants rated satisfaction highly, via both the quantitative satisfaction and evaluation questionnaire and the exit interviews, indicating that a full-scale RCT would be well received by university students. Participants were satisfied with the two-week check in points (94.4% satisfaction across the two groups), thus this should be retained in the large-scale RCT study protocol. If the budget is limited, a two-week automated or internet-based check in could be utilized, with face-to-face meetings once per month to cut resource costs. Additional changes to consider come from the exit interviews; including making the intervention materials available online to increase participant access to their goals and plans, as well as increasing accountability by using phone reminders to trigger adherence to PA plans.

The secondary outcome measure of PA change showed promising directional trends, as hypothesized. The main effect was large for the comparison of LSI scores from baseline to six weeks. This suggests that both the direct intervention and the indirect identity intervention materials may be effective in increasing overall PA. This finding supports the efficacy of both groups’ intervention materials; including emphasis on perceived competence, perceived enjoyment and BCTs including planning, goal-setting, problem solving, and self-monitoring. Bray et al. (2011), and Kwan et al. (2013) both saw a decrease in overall PA levels of their participants at six and eight weeks respectively, yet still recommended their interventions for full-scale trials based on their feasibility data. Therefore, positive trends in PA data in this research strongly support the recommendation for a full-scale RCT, in addition to the high feasibility ratings.

Finally, it was hypothesized that identity change would show upwards trends in both intervention groups, with larger changes observed in the direct intervention group. The overall effect size from baseline to 6 weeks was large, meaning both groups increased their exercise identity scores. This is promising for a larger trial and supports the malleability of the identity construct, as well as the efficacy of both direct and indirect interventions to change identity. However, the interaction effect was small (*η^2^*^ ^= 0.03), meaning the effect of the direct intervention materials on exercise identity scores was negligible in comparison to the standard intervention group. It is important to note that the direct intervention group had much higher identity scores at baseline than the indirect group and this could have compromised our analyses of change trends. Thus, the dual pathway model to changing identity as suggested in the introduction may still hold, but it is not clear which path was more successful due to the nature of a feasibility study.

For the full-scale RCT, in addition to a larger participant pool, a longer intervention period is needed in order to accurately determine the effect of this study on PA. A period of time that is longer than one university semester is recommended to avoid inflated results due to a short term intervention (Plotnikoff et al., 2015). Additionally, it is recommended to include a follow-up period in order to assess the efficacy of the intervention once participants are no longer meeting with the instructor. A third arm – a true control group who only completes measures but receives no intervention would also be useful to explore both interventions.

This study is not without its limitations. First and foremost, the participant sample, although self-selected as being under PA guidelines (150 min MVPA per week), was already quite active at baseline. Additionally, commensurate with an active sample, the mean exercise identity scores at baseline were in the ‘medium’ range. Overall, this sample was already participating at PA levels sufficient for health benefits. In addition, participants were overwhelmingly (72.2%) female which may limit the generalizability of this programme to male participants. Furthermore, only 11/18 participants opted in for the exit interviews. It is possible the other 7/18 participants may have had largely different responses and overall satisfaction levels. However, 18/18 participants completed the satisfaction and evaluation questionnaire which also yielded high scores. Lastly, the 6-week time frame in this study is short. This short time frame may be partially responsible for the high retention rates, and it is unclear if a longer study presenting the same materials would have the same high retention. The retention rate in this study, while promising, is uncertain when situated in terms of a longer trial.

In conclusion, these results support the need to expand standard, social-cognitive theories to include other processes such as identity (Perrier, Sweet, Strachan, & Latimer-Cheung, 2012; Rhodes et al., 2016; Rise, Sheeran, & Hukkelberg, 2010; Strachan, Woodgate, Brawley, & Tse, 2005). Despite the known relationship between identity and PA, there has been little research on the feasibility of intervening upon identity as a means to increase PA. The current study examined the feasibility of identity intervention among university students via two routes: an indirect route that follows attempting to help people self-regulate physical activity and build competence until identity naturally forms, and a direct route whereby identity is formed via intervention material that attempts to expedite the process. Feasibility results indicated high retention and satisfaction rates, but difficulties with recruitment, as well as promising preliminary results in terms of increased PA and increased exercise identity scores in both groups. These results support recommendations for a full-scale RCT, with modifications to handle recruitment difficulties, as well as minor changes to study design including increased accountability through phone reminders and increased emphasis on counselling sessions over print materials.

## Supplementary Material

Supplemental Material

## References

[CIT0001] Ajzen, I. (1991). The theory of planned behavior. *Organizational Behavior and Human Decision Processes*, *50*(2), 179–211. doi: 10.1016/0749-5978(91)90020-

[CIT0002] Amireault, S., & Godin, G. (2015). The Godin-Shephard Leisure-time physical activity questionnaire: Validity evidence Supporting its Use for Classifying healthy adults into active and Insufficiently active Categories. *Perceptual and Motor Skills*, *120*(2), 604–622. doi: 10.2466/03.27.PMS.120v19x7 25799030

[CIT0003] Anderson, D. F., & Cychosz, C. (1994). Development of an Exercise Identity Scale. *Perceptual and Motor Skills*, *78*(3), 747–751. doi: 10.2466/pms.1994.78.3.747 8084685

[CIT0004] Arigo, D. (2015). Promoting physical activity among women using wearable technology and online social connectivity: A feasibility study. *Health Psychology and Behavioral Medicine*, *3*(1), 391–409. doi: 10.1080/21642850.2015.1118350

[CIT0005] Bandura, A. (1998). Health promotion from the perspective of social cognitive theory. *Psychology & Health*, *13*(4), 623–649. doi: 10.1080/08870449808407422

[CIT0006] Bem, D. J. (1972). Self-perception theory. *Advances in Experimental Social Psychology*, *6*, 1–62. doi:10.1016/S0065-2601(08)60024-6

[CIT0007] Berry, T. R., Strachan, S. M., & Verkooijen, K. T. (2013). The relationship between exercise schema and identity. *International Journal of Sport and Exercise Psychology*, *12*(1), 49–63. doi: 10.1080/1612197X.2013.775742

[CIT0008] Bowen, D. J., Kreuter, M., Spring, B., Cofta-Woerpel, L., Linnan, L., Weiner, D., … Fernandez, M. (2009). How we design feasibility studies. *American Journal of Preventive Medicine*, *36*(5), 452–457. doi: 10.1016/j.amepre.2009.02.002 19362699 PMC2859314

[CIT0009] Bray, S. R., Beauchamp, M. R., Latimer, A. E., Hoar, S. D., Shields, C. A., & Bruner, M. W. (2011). Effects of a print-mediated intervention on physical activity during transition to the first year of university. *Behavioral Medicine*, *37*(2), 60–69. doi: 10.1080/08964289.2011.571306 21660774

[CIT0010] Bray, S. R., & Born, H. A. (2004). Transition to university and vigorous physical activity: Implications for health and Psychological well-being. *Journal of American College Health*, *52*(June), 181–188. doi: 10.3200/JACH.52.4.181-188 15018429

[CIT0011] Burke, P. J. (2016). Identity Change. *Social Psychology Quarterly*, *69*(1), 81–96. doi: 10.1177/019027250606900106

[CIT0012] Canadian Society for Exercise Physiologists. (2018). Canadian Physical Activity Guidelines - for adults. Retrieved from www.csep.ga/guidelines.

[CIT0013] Canning, C. G., Allen, N. E., Dean, C. M., Goh, L., & Fung, V. S. C. (2012). Home-based treadmill training for individuals with Parkinson’s disease: A randomized controlled pilot trial. *Clinical Rehabilitation*, *26*(9), 817–826. doi: 10.1177/0269215511432652 22257506

[CIT0014] Cohen, J. (1992). A power primer. *Psychological Bulletin*, *112*(1), 155–159. doi: 10.1037//0033-2909.112.1.155 19565683

[CIT0015] Eldridge, S. M., Chan, C. L., Campbell, M. J., Bond, C. M., & Hopewell, S. (2016). CONSORT 2010 statement : extension to randomised pilot and feasibility trials the consolidated standards of reporting trials (CONSORT) statement reporting of randomised controlled an extension to that statement for. *BMJ: British Medical Journal*, *355*, 1–29. doi: 10.1136/bmj.i5239

[CIT0016] Festinger, L. (2016). A theory of social comparison processes. *Human Relations*, *7*(2), 117–140. doi: 10.1177/001872675400700202

[CIT0017] Field, A. P, Field, Z, & Miles, J. (2013). *Discovering statistics using R*. Los Angeles: Sage.

[CIT0018] Fishbein, M., Triandis, H. C., & Eichler, A. (2001). Factors influencing behavior and behavior change. In A. Baum & T.A. Revenson (Eds.), *Handbook of health psychology* (pp. 3–17). New Jersey, Mahwah: Lawrence Erlbaum Associates.

[CIT0019] Forbes, C. C., Blanchard, C. M., Mummery, W. K., & Courneya, K. S. (2015). Feasibility and preliminary efficacy of an online intervention to increase physical activity in Nova Scotian cancer survivors: A randomized Controlled trial. *JMIR Cancer*, *1*(2), e12. doi: 10.2196/cancer.4586 28410166 PMC5367676

[CIT0020] Gardner, B., De Bruijn, G. J., & Lally, P. (2011). A systematic review and meta-analysis of applications of the self-report habit index to nutrition and physical activity behaviours. *Annals of Behavioral Medicine*, *42*(2), 174–187. doi: 10.1007/s12160-011-9282-0 21626256

[CIT0021] Gionet, N. J, & Godin, G. (1989). Self-reported exercise behaviour of employees: A validity study. *Journal of Occupational Medicine*, *31*, 969–973. 2614536 10.1097/00043764-198912000-00007

[CIT0022] Godin, G, & Shephard, R. J. (1997). *Godin Leisure-Time Exercise Questionnaire. Medicine and Science in Sports and Exercise* (pp. S36–S38). doi: 10.2466/03.27.PMS.120v19x7

[CIT0023] Gyurcsik, N. C., Bray, S. R., & Brittain, D. R. (2004). Coping with barriers to vigorous physical activity during transition to university. *Family & Community Health*, *27*(2), 130–142. doi: 10.1097/00003727-200404000-00006 15596980

[CIT0024] Hagger, M. S., & Chatzisarantis, N. L. D. (2014). An integrated behavior change model for physical activity. *Exerc. Sport Sci. Rev*, *42*(2), 62–69. doi: 10.1249/JES.0000000000000008 24508739

[CIT0025] Hallal, P. C., Andersen, L. B., Bull, F. C., Guthold, R., Haskell, W., & Ekelund, U. (2012). Physical activity 1 Global physical activity levels: Surveillance progress, pitfalls, and prospects. *The Lancet*, *380*, 247–257. doi: 10.1016/S0140-6736(12)60646-1 22818937

[CIT0026] Jackson, N., & Waters, E. (2005). Criteria for the systematic review of health promotion and public health interventions. *Health Promotion International*, *20*(4), 367–374. doi: 10.1093/heapro/dai022 16169885

[CIT0027] Kendzierski, D., Furr, R. M., & Schiavoni, J. (1998). Physical activity self-definitions: Correlates and perceived criteria. *Journal of Sport & Exercise Psychology*, *20*, 176–193.

[CIT0028] Kwan, M., Faulkner, G., & Bray, S. (2013). Evaluation of active transition, a website-delivered physical activity intervention for university students: Pilot study. *Journal of Medical Internet Research*, *15*(5), 1–9. doi: 10.2196/resprot.2099 PMC3650928 23649858

[CIT0029] Kwasnicka, D., Dombrowski, S. U., White, M., & Sniehotta, F. (2016). Theoretical explanations for maintenance of behaviour change: A systematic review of behaviour theories. *Health Psychology Review*, *10*(3), 277–296. doi: 10.1080/17437199.2016.1151372 26854092 PMC4975085

[CIT0030] Lee, I. M., Shiroma, E. J., Lobelo, F., Puska, P., Blair, S. N., Katzmarzyk, P. T., … Wells, J. C. (2012). Effect of physical inactivity on major non-communicable diseases worldwide: An analysis of burden of disease and life expectancy. *The Lancet*, *380*(9838), 219–229. doi: 10.1016/S0140-6736(12)61031-9 PMC3645500 22818936

[CIT0031] Linnan, L., & Steckler, A. (2002). *An Overview*. (A. Steckler & L. Linnan, Eds.), *Process evaluation for public health interventions and research*. San Francisco: Jossey-Bass. doi: 10.1016/j.evalprogplan.2003.09.006

[CIT0032] Michie, S., Abraham, C., Whittington, C., McAteer, J., & Gupta, S. (2009). Effective techniques in healthy eating and physical activity interventions: A meta-regression. *Health Psychology*, *28*(6), 690–701. doi: 10.1037/a0016136 19916637

[CIT0033] Michie, S., Richardson, M., Johnston, M., Abraham, C., Francis, J., Hardeman, W., … Wood, C. E. (2013). The behavior change technique taxonomy (v1) of 93 hierarchically clustered techniques: Building an international consensus for the reporting of behavior change interventions. *Annals of Behavioral Medicine*, *46*(1), 81–95. doi: 10.1007/s12160-013-9486-6 23512568

[CIT0034] Moore, G. F., Audrey, S., Barker, M., Bond, L., Bonell, C., Hardeman, W., … Baird, J. (2015). Process evaluation of complex interventions: Medical research council guidance. *BMJ*, *350*(h1258), 1–7. doi: 10.1136/bmj.h1258 PMC4366184 25791983

[CIT0035] Perrier, M. J., Sweet, S. N., Strachan, S. M., & Latimer-Cheung, A. E. (2012). I act, therefore I am: Athletic identity and the health action process approach predict sport participation among individuals with acquired physical disabilities. *Psychology of Sport and Exercise*, *13*(6), 713–720. doi: 10.1016/j.psychsport.2012.04.011

[CIT0036] Peterson, R. A., & Merunka, D. R. (2014). Convenience samples of college students and research reproducibility. *Journal of Business Research*, *67*(5), 1035–1041. doi: 10.1016/j.jbusres.2013.08.010

[CIT0037] Plotnikoff, R. C., Costigan, S. A., Williams, R. L., Hutchesson, M. J., Kennedy, S. G., Robards, S. L., … Germov, J. (2015). Effectiveness of interventions targeting physical activity, nutrition and healthy weight for university and college students: A systematic review and meta-analysis. *International Journal of Behavioral Nutrition and Physical Activity*, *12*(1), 1–10. doi: 10.1186/s12966-015-0203-7 PMC4393577 25890337

[CIT0038] Prochaska, J. O., & DiClemente, C. C. (1982). Transtheoretical therapy: Toward a more integrative model of change. *Psychotherapy: Theory, Research & Practice*, *19*(3), 276–288. doi: 10.1037/h0088437

[CIT0039] Quinlan, A., Rhodes, R. E., Blanchard, C. M., Naylor, P.-J., & Warburton, D. E. R. (2015). Family planning to promote physical activity: A randomized controlled trial protocol. *BMC Public Health*, *15*(1), 1011. doi: 10.1186/s12889-015-2309-x 26437939 PMC4594902

[CIT0040] Rebar, A. L., Dimmock, J. A., Jackson, B., Rhodes, R. E., Kates, A., Starling, J., & Vandelanotte, C. (2016). A systematic review of the effects of non-conscious regulatory processes in physical activity. *Health Psychology Review*, 1–13. doi: 10.1080/17437199.2016.1183505 27118430

[CIT0041] Rebar, A. L., Stanton, R., Geard, D., Short, C., & Duncan, M. J. (2015). A meta-meta-analysis of the effect of physical activity on depression and anxiety in non-clinical adult populations. *Health Psychology Review*, *9*(3), 366–378. doi: 10.1080/17437199.2015.1022901 25739893

[CIT0042] Rhodes, R. (2017). The Evolving understanding of physical activity behavior: A multi-process action control approach. *Advances in Motivation Science* (Vol. 4). Elsevier Ltd. doi: 10.1016/bs.adms.2016.11.001

[CIT0043] Rhodes, R. E., Gray, S. M., & Husband, C. (2018). Experimental manipulation of affective judgments about physical activity: A systematic review and meta-analysis of adults. *Health Psychology Review*, 1–17. doi: 10.1080/17437199.2018.1530067 30261826

[CIT0044] Rhodes, R. E., Janssen, I., Bredin, S. S. D., Warburton, D. E. R., & Bauman, A. E. (2017). Physical activity : health impact, prevalence, correlates and interventions. *Psychology & Health*, *32*(8), 942–975. doi: 10.1080/08870446.2017.1325486 28554222

[CIT0045] Rhodes, R. E., Kaushal, N., & Quinlan, A. (2016). Is physical activity a part of who I am? A review and meta-analysis of identity, schema and physical activity. *Health Psychology Review*, *10*(2), 204–225. doi: 10.1080/17437199.2016.1143334 26805431

[CIT0046] Rhodes, R. E., Naylor, P. J., & McKay, H. A. (2010). Pilot study of a family physical activity planning intervention among parents and their children. *Journal of Behavioral Medicine*, *33*(2), 91–100. doi: 10.1007/s10865-009-9237-0 19937106

[CIT0047] Ries, F., Hein, V., Pihu, M., & Armenta, J. M. S. (2012). Self-identity as a component of the theory of planned behaviour in predicting physical activity. *European Physical Education Review*, *18*(3), 322–334. doi: 10.1177/1356336X12450792

[CIT0048] Rise, J., Sheeran, P., & Hukkelberg, S. (2010). The role of self-identity in the theory of planned behavior : A meta-analysis. *Journal of Applied Social Psychology*, *40*(5), 1085–1105, Q4. doi: 10.1111/j.1559-1816.2010.00611.x

[CIT0049] Schulz, K. F., Altman, D. G., Moher, D., & Group, C. (2010). Academia and clinic annals of internal medicine CONSORT 2010 statement : Updated guidelines for reporting parallel group randomized trials. *Annals of Internal Medicine*, *1996*(14), 727–732. doi: 10.7326/0003-4819-152-11-201006010-00232 20335313

[CIT0050] Sheeran, P., Gollwitzer, P. M., Bargh, J. A., Gollwitzer, P. M., & Bargh, J. A. (2013). Nonconscious processes and health nonconscious processes and health. *Health Psychology*, *32*(5), 460–473. doi: 10.1037/a0029203 22888816

[CIT0051] Skår, S., Sniehotta, F. F., Molloy, G. J., Prestwich, A., & Araújo-Soares, V. (2011). Do brief online planning interventions increase physical activity amongst university students? A Randomised Controlled Trial. *Psychology and Health*, *26*(4), 399–417. doi: 10.1080/08870440903456877 20830646

[CIT0052] Stadler, G., Oettingen, G., & Gollwitzer, P. M. (2009). Physical Activity in Women. Effects of a self-regulation intervention. *American Journal of Preventive Medicine*, *36*(1), 29–34. doi: 10.1016/j.amepre.2008.09.021 18977113

[CIT0053] Stets, J. E., & Burke, P. J. (2000). Identity theory and social identity theory. *Social Psychology Quarterly*, *63*(3), 224–237. doi: 10.2307/2695870

[CIT0054] Strachan, S. M., & Brawley, L. R. (2008). Reactions to a perceived challenge to identity: A focus on exercise and healthy eating. *Journal of Health Psychology*, *13*(5), 575–588. doi: 10.1177/1359105308090930 18519432

[CIT0055] Strachan, S. M., Woodgate, J., Brawley, L., & Tse, A. (2005). The relationship of self-efficacy and self-identity to long-term maintenance of vigorous physical activity. *Journal of Applied Biobehavioral Research*, *10*(2), 98–112. doi: 10.1111/j.1751-9861.2005.tb00006.x

[CIT0056] Strack, F., & Deutsch, R. (2004). Reflective and impulsive determinants of social behavior. *Personality and Social Psychology Review : An Official Journal of the Society for Personality and Social Psychology, Inc,*, *8*(3), 220–247. doi: 10.1207/s15327957pspr0803_1 15454347

[CIT0057] Stryker, S., & Burke, P. (2000). The past, present, and future of an identity theory author (s): Sheldon Stryker and Peter J. Burke Source : social psychology Quarterly, Vol. 63, No. 4, special millenium issue on the state of sociological social psychology (Dec ., 2000). *Social Psychology*, *63*(4), 284–297. doi: 10.2307/2695840

[CIT0058] Van Oort, C., Tupper, S. M., Rosenberg, A. M., Farthing, J. P., & Baxter-Jones, A. D. (2013). Safety and feasibility of a home-based six week resistance training program in juvenile idiopathic arthritis. *Pediatric Rheumatology*, *11*(1). doi: 10.1186/1546-0096-11-46 PMC3878188 24359015

[CIT0059] Warburton, D. E.R., Nicol, C.W., & Bredin, S.S.D. (2006). Health benefits of physical activity: The evidence. *Canadian Medical Association Journal*, *174*(6), 801–809. doi: 10.1503/cmaj.051351 16534088 PMC1402378

[CIT0060] West, R. (2009). The multiple facets of cigarette addiction and what they mean for encouraging and helping smokers to stop. *Journal of Chronic Obstructive Pulmonary Disease*, *6*(4), 277–283. doi: 10.1080/15412550903049181 19811387

[CIT0061] Williams, D. M., & Evans, D. R. (2014). Current emotion research in health behavior science. *Emotion Review*, *6*(3), 277–287. doi: 10.1177/1754073914523052 PMC12975116 41815188

