# Supplementary material for: A feasibility randomized trial of an identity-based physical activity intervention among university students
Source: Health Psychol Behav Med. 2019 Apr 10;7(1):128–46. doi: 10.1080/21642850.2019.1600407 (PMC8114372; doi:10.1080/21642850.2019.1600407)
Supplement: Supplemental Material [file RHPB_A_1600407_SM3729.pdf]

Exit Interview Questions

1. Tell me about how you felt about the study?
2. Did you find the meeting sessions with the research investigator useful?
3. Have you been able to incorporate the strategies provided in the workbooks and the ones you brainstormed during the one-on-one sessions? If so, how? If not, how come?
4. Did you find the check-in sessions helpful?
5. What was your favorite part?
6. What was your least favorite part?
7. What would you change?
8. Do you feel like your participation in the study helped you with your physical activity participation?
9. Do you feel like your participation in the study helped you create an identity for physical activity?
10. Do you feel passionate about your physical activity?
11. Do you have any other comments?

For Peer Review Only

Intervention Materials - *Indirect Intervention* Group

Session 1

Physical Activity Benefits and Goal Setting

For Peer Review

Physical Activity Guidelines

The Canadian Society for Exercise Physiology has physical activity guidelines for people of all ages. Adults aged 18-64 years should be getting **150 minutes of moderate to vigorous physical activity per week**.

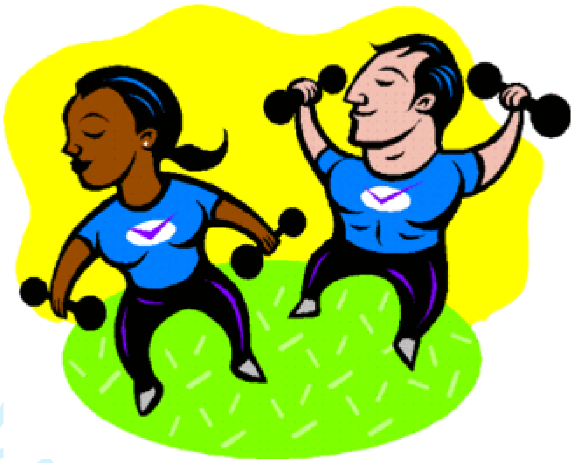

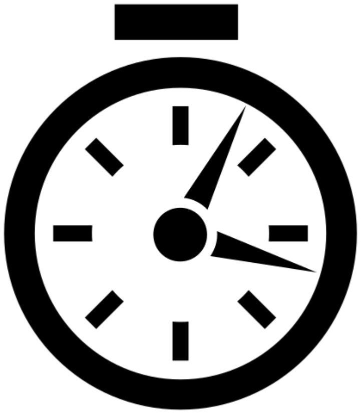

Physical Benefits of Regular Physical Activity

150 minutes per week of activity can help reduce your risk of:

- Cardiovascular disease
- Heart disease
- Diabetes
- Certain types of cancer
- Stroke
- Hypertension
- Obesity
- Osteoporosis

Other Benefits of Regular Physical Activity

- Increased strength
- Improved quality of life
- Reduction of depressive symptoms
- Reduced stress

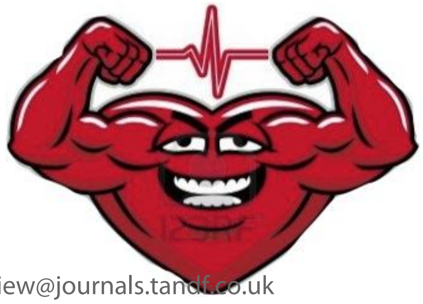

Session 1

Activities

Activity 1:

In the space to the right, **brainstorm** at least 3 ideas of physical activity you could see yourself doing.

Activity 2:

In the space to the right, **set a physical activity goal** for the coming two weeks, using the activities you have brainstormed above.

Activity 3:

In the space to the right, **plan how** you will achieve your physical activity goal in the coming two weeks.

For Peer Review Only

Session 2

Check In and Re-set Goals

For Peer Review

Check in

Did you meet your goals?

In the space to the right, record your physical activity participation in the past two weeks.

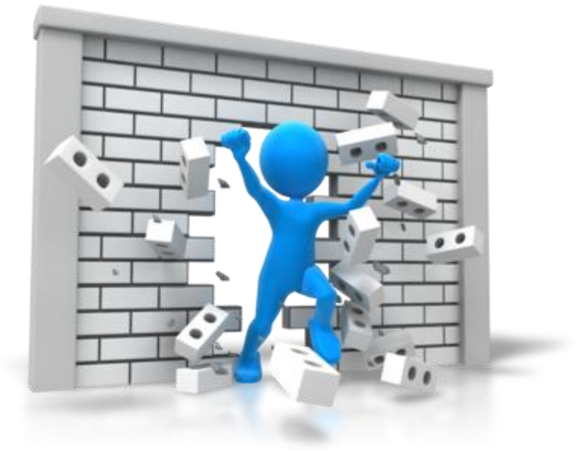

Barriers

Barriers are things that prevented, or made it more difficult for you to reach your physical activity goals.

In the space below, list some barriers you encountered over the past two weeks.

Self-Monitoring

Being aware of your behaviour and adjusting as necessary. Anytime you reflect on what you have done you are self-monitoring.

Example: Journal, calendar, apps

Session 2

Activities

Activity 1:

In the space to the right, re-set your physical activity **goals** based on what you experienced in the past two weeks.

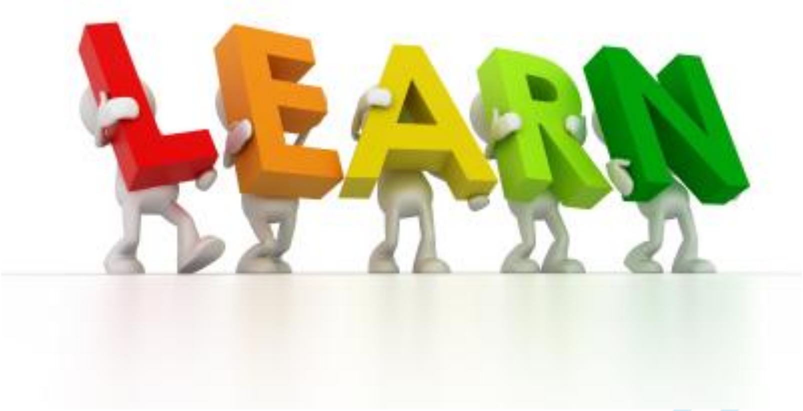

Activity 2:

In the space to the right, re-vamp your physical activity **plan** based on what you experienced in the past two weeks.

Session 3

Check In and Re-set Goals

Check in

Did you meet your goals?

In the space to the right, record your physical activity participation in the past two weeks.

Activity 1:

In the space to the right, re-set your physical activity **goals** based on what you experienced in the past two weeks.

Activity 2:

In the space to the right, re-vamp your physical activity **plan** based on what you experienced in the past two weeks.

For Peer Review

Direct Intervention Group

Session 1

Physical Activity Benefits and Goal Setting

Physical Activity Guidelines

The Canadian Society for Exercise Physiology has physical activity guidelines for people of all ages. Adults aged 18-64 years should be getting **150 minutes of moderate to vigorous physical activity per week**.

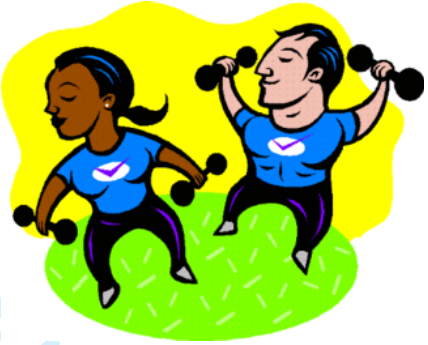

Physical Benefits of Regular Physical Activity

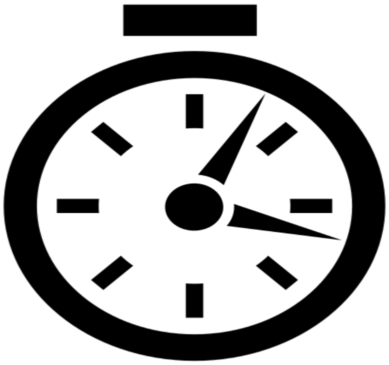

150 minutes per week of activity can help reduce your risk of:

- Cardiovascular disease
- Heart disease
- Diabetes
- Certain types of cancer
- Stroke
- Hypertension
- Obesity
- Osteoporosis

Other Benefits of Regular Physical Activity

- Increased strength
- Improved quality of life
- Reduction of depressive symptoms
- Reduced stress

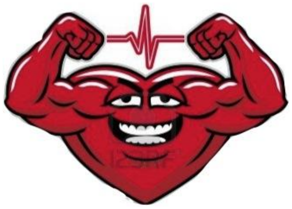

Session 1

Activities and Exercise Identity

Activity 1:

In the space to the right, **brainstorm** at least 3 ideas of physical activity you could see yourself doing.

It is important to pick activities that are *fun* and you think you will be *good at*.

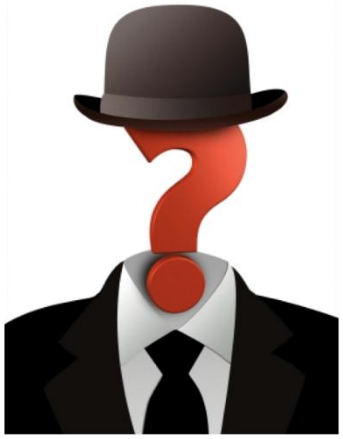

Exercise Identity

Having an exercise identity is when physical activity participation is part of who you are as a person. Exercise identity is correlated with the following:

- Frequency of exercise
- Duration of exercise
- Intensity of exercise

Activity 2:

In the space to the right, create a physical activity **goal** for the coming two weeks, using the activities you have brainstormed above.

Session 1

Activities

Activity 3:

In the space to the right, **plan how** you will achieve your physical activity goal in the coming two weeks.

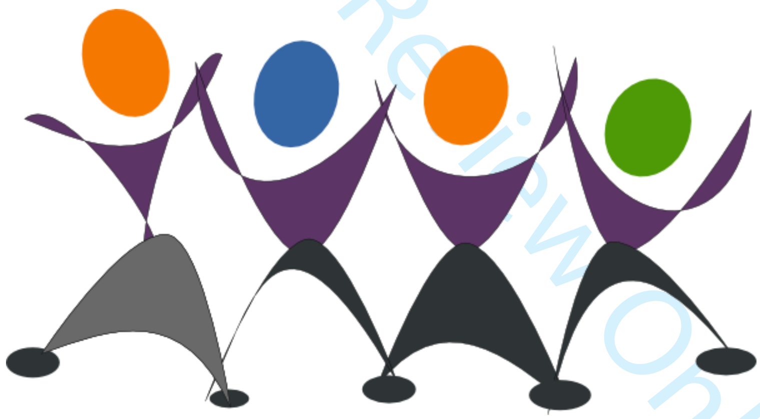

Activity 4:

In the space to the right, **brainstorm** how you will make your physical activity participation fun. If you are having fun, it will be easier to motivate yourself to achieve your physical activity goals.

For Peer Review Only

Session 2

Check In and Re-set Goals

Check in

Did you meet your goals?

In the space to the right, record your physical activity participation in the past two weeks.

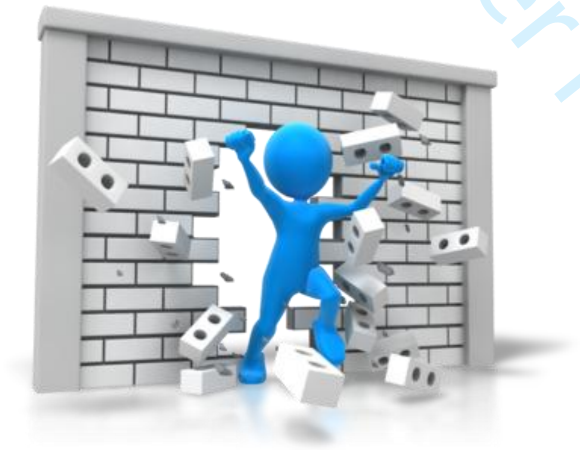

Barriers

Barriers are things that prevented, or made it more difficult for you to reach your physical activity goals.

In the space below, list some barriers you encountered over the past two weeks.

Self-Monitoring

Being aware of your behaviour and adjusting as necessary. Anytime you reflect on what you have done you are self-monitoring.

Example: Journal, calendar, app

Session 2

Priorities and Self-Talk

Priorities

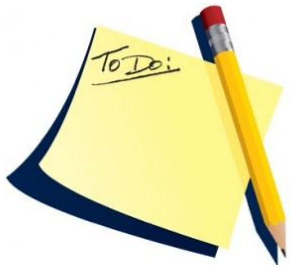

You hold multiple identities, and therefore have competing priorities when it comes to allocating your time, money, and effort.

In theory, the things you spend the most time, money, and effort on should correlate with what you identify most strongly.

Activity 1:

In the space below, list your top 5 priorities in order.

Activity 2:

In the space below, list the top 5 things you spend time, money, and effort on.

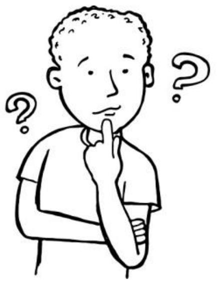

Self-Talk

In the face of a barrier, it can be helpful to remind yourself that participating in physical activity is a part of who you are. In the space to the right, create a phrase to say to yourself when you are facing a barrier.

Session 2

Activities and Rules

Activity 3:

Considering your priorities, re-set your physical activity **goals** based on what you experienced in the past two weeks.

Activity 4:

In the space to the right, re-vamp your physical activity **plan** based on what you experienced in the past two weeks.

Also, consider the barriers you have faced these past two weeks when creating your plan.

For Peer Review Only

Sacrifice and Rules

One of the ways to build an exercise identity is to create **rules** around your physical activity participation.

For example: when you go to yoga there are rules of the studio and un-written rules about the attire.

Activity 5:

In the space to the right, create a rule around your physical activity participation.

Session 3

Check In and Re-set Goals

Check in

Did you meet your goals?

In the space to the right, record your physical activity participation in the past two weeks.

Activity 1:

In the space to the right, re-set your physical activity **goals** based on what you experienced in the past two weeks.

Activity 2:

In the space to the right, re-vamp your physical activity **plan** based on what you experienced in the past two weeks.

For Peer Review Only

Session 3

Environment and Cues

Environment

Your environment can help you participate in physical activity.

- Habits are attached to environmental cues
- Creating an environment that is conducive to physical activity participation

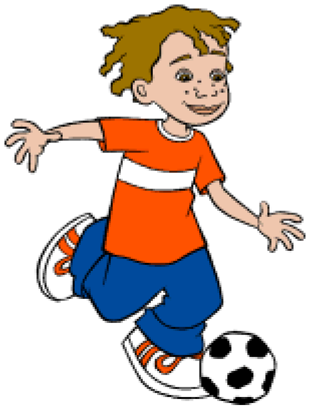

Activity 3:

In the space to the right, brainstorm some cues in your environment that you could attach physical activity to.

Example: going to the gym on your drive home from school.

Activity 4:

In the space to the left, brainstorm ways you could change your environment to make it easier to do physical activity.

Example: runners and athletic clothes laid out the night before a morning run.

Session 3

Symbolic Representation

Symbolism

The way you represent yourself to others will impact how you perceive yourself as well. Having an identity means showing it off in some way.

- Clothes
- Hairstyle
- Social media presence
- Decoration of your personal space

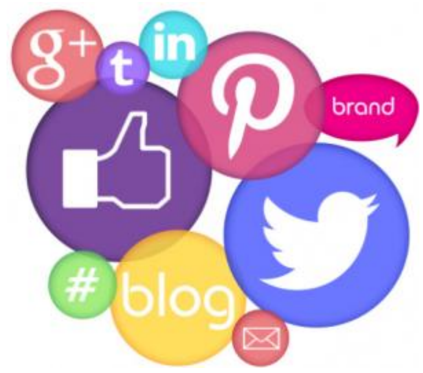

Activity 5:

In the space below, record how you think others perceive you.

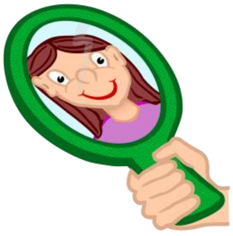

Activity 6:

In the space below, pick one way you can "amp up" your physical activity identification.
